# Current indications for insertion of rotary pumps revisited

**DOI:** 10.21542/gcsp.2016.25

**Published:** 2016-09-30

**Authors:** Leslie W Miller

**Affiliations:** Cardiology Division, Washington Hospital Center, 110 Irving St NW, Room NA-1101, Washington DC 20010, USA

Cardiovascular disease (CVD) remains the leading cause of death as well as morbidity in the world^1–3^. While there has been continued progress in reducing the mortality of most forms of CVD, the prevalence, as well as mortality and morbidity from heart failure, continues to increase, making it a major health care problem world wide. This increase in prevalence is in part due to the correlation of increasing CVD with advancing age, but also to improved diagnostics and earlier detection as well as improved interventions for acute MI and medical management. Despite these advances, an increasing number of patients become unresponsive to therapy and progress to the advanced stage, which has a mortality as high as 70–80% at one year.

The only approved treatment options for advanced HF are heart transplantation and mechanical circulatory support with use of left ventricular assist devices (LVADs). Transplantation has been severely limited by a chronic shortage of donors, while LVAD therapy has experienced significant growth due to major advances in the technology, primarily the switch from pulsatile to rotary design pumps. While initially employed for only those in profound shock, the Holy Grail in the field has been to extend its use into less sick patients, especially those beyond transplant eligibility, so-called Destination Therapy, where the greatest potential growth exists, especially within aging populations.

In this issue of the journal, Kirklin^4^ examines the data available to address this important question of whether the field is ready to extend LVAD therapy to a less sick, ambulatory population of patients with heart failure. The author addresses this question by reviewing all the patients who have undergone LVAD placement for either Bridge-to-Transplant (BTT) or Destination Therapy (DT) indications primarily in the U.S., that have been captured in a very well maintained registry called INTERMACS, which covers more than 10,000 patients. The author has provided a substantial number of figures from that registry to provide the reader with a clear overview of the actual data used to support his review. As he points out, the registry data has made it very clear that survival is directly proportional to the severity of heart failure/shock at the time of implant, being lowest in the sickest patients. Awareness of this data has led to a progressive decrease in the percentage of patients who are in true or borderline shock at the time of implant in both groups. However, currently only 20% of the typically more elective implants in the DT population were ambulatory at the time of surgery.

The author points out that the major impediment to the expansion of LVAD therapy is the relatively high incidence of adverse effects and complications including bleeding, pump thrombosis, and stroke which are higher in the rotary pumps than was seen with pulsatile design LVADs. The improvements in outcomes, especially survival, with the conversion to rotary design pumps led many experts in the field to believe that there was now sufficient evidence to have equipoise about whether LVADs had reached a stage of development that outcomes could equal that with medical therapy and a randomized trial was plausible. However, the several reports of increasing prevalence of pump thrombosis and device replacement led to halting the initiation of the long awaited NIH sponsored randomized trial of LVAD vs medical therapy, called REVIVEIT before it ever began enrollment. Subsequent data has shown a decline in these problems with better long-term management of anticoagulation. There is little data to suggest that there is any significant difference in the problems of pump thrombosis between the Heartmate and Heartware devices, and thus represents a broad criticism of this generation of LVADs. Anticoagulation is perhaps the Achilles heel of LVAD therapy, with a challenging balance between pump thrombosis and bleeding, particularly in the GI track. This is an area of significant focus currently.

It is also clear that outcomes are strongly influenced by the presence of comorbidities in the patient at the time of LVAD implant, with the presence of significant renal dysfunction and right heart failure having the greatest impact. The author chose an unusual case-based approach to demonstrate the dilemma of extending into less sick patients. The first case involved a patient with few comorbidities and their survival equaled transplantation. The other patient had several comorbidities that led to an estimated survival that would be inferior to transplantation.

Another important metric that will influence the expansion of LVAD therapy into less sick patients is cost-effectiveness. There is an increasing use of this metric to judge the financial feasibility of this therapy, but this subject was not addressed in the review by Kirklin. One of the challenging factors to weight in the overall assessment of the benefit with LVADs is quality of life. There is fairly substantive data to support a significant improvement in self assessed quality of life out to two years post implant with LVADs.

The review by Kirklin provides an objective look at the current status of the data that will be used to justify an expansion of LVAD therapy into less sick heart failure patients. The conclusions presented provide a very balanced assessment of the issues that need to be resolved, but it seems that several current problems need to be resolved before the expansion into less sick patients will be ready for clinical trial in the foreseeable future.
